# Risk factors for central venous catheter-associated deep venous thrombosis in pediatric critical care settings identified by fusion model

**DOI:** 10.1186/s12959-022-00378-y

**Published:** 2022-04-12

**Authors:** Haomin Li, Yang Lu, Xian Zeng, Yuqing Feng, Cangcang Fu, Huilong Duan, Qiang Shu, Jihua Zhu

**Affiliations:** 1 The Children’s Hospital of Zhejiang University School of Medicine and National Clinical Research Center for Child Health, Hangzhou, China; 2 Clinical Data Center, The Children’s Hospital, Zhejiang University School of Medicine, 3333 Binsheng Road, 310052 Hangzhou, China; 3 The College of Biomedical Engineering and Instrument Science, Zhejiang University, Hangzhou, China; 4 Department of Nursing, The Children’s Hospital, Zhejiang University School of Medicine, 3333 Binsheng Road, 310052 Hangzhou, China

**Keywords:** Central venous catheter, Deep venous thrombosis, Pediatric critical care, Machine learning, Risk factors

## Abstract

**Background:**

An increase in the incidence of central venous catheter (CVC)-related thrombosis (CRT) has been reported in pediatric intensive care patients over the past decade. Risk factors for the development of CRT are not well understood, especially in children. The study objective was to identify potential clinical risk factors associated with CRT with novel fusion machine learning models.

**Methods:**

Patients aged 0–18 who were admitted to intensive care units from December 2015 to December 2018 and underwent at least one CVC placement were included. Two fusion model approaches (stacking and blending) were used to build a better performance model based on three widely used machine learning models (logistic regression, random forest and gradient boosting decision tree). High-impact risk factors were identified based on their contribution in both fusion artificial intelligence models.

**Results:**

A total of 478 factors of 3871 patients and 3927 lines were used to build fusion models, one of which achieved quite satisfactory performance (AUC = 0.82, recall = 0.85, accuracy = 0.65) in 5-fold cross validation. A total of 11 risk factors were identified based on their independent contributions to the two fusion models. Some risk factors, such as D-dimer, thrombin time, blood acid-base balance-related factors, dehydrating agents, lymphocytes and basophils were identified or confirmed to play an important role in CRT in children.

**Conclusions:**

The fusion model, which achieves better performance in CRT prediction, can better understand the risk factors for CRT and provide potential biomarkers and measures for thromboprophylaxis in pediatric intensive care settings.

**Supplementary information:**

The online version contains supplementary material available at 10.1186/s12959-022-00378-y.

## Background

Central venous catheters (CVCs) have revolutionized the care of patients requiring long-term venous access. The introduction of CVC in pediatric intensive care has been one of the important modalities in the improvement of quality of care in critical patients [1]. Despite these advantages, more than 15% of patients receiving CVC catheter treatment could have complications, such as mechanical complications are reported to occur in 5 to 19% of patients, infectious complications in 5 to 26%, and CVC-associated deep venous thrombosis (CADVT) in 2 to 26 precent [2, 3], which lead to an increased length of hospital stay and medical costs. CADVT constitutes 10% of all deep venous thrombosis (DVT) in adults and 50–80% of all DVTs among children [4], and nearly all DVT-related deaths in children are associated with CVCs [5]. In newborns, approximately 90% of venous thrombosis is related to CVC [6]. In pediatric patients, the presence of a CVC is the single most common risk factor for venous thromboembolism (VTE) [7, 8]. With the increasing use of CVCs, the incidence of CADVT has been on the rise. A significant increase in the rate by 30–70% has been reported among hospitalized children over the last 2 decades [9, 10].

There is a compelling need for comprehensive studies that can identify and evaluate the modifiable risk factors for CADVT using routine clinical data to offer opportunities to decrease thrombosis and improve outcomes. Mondal and colleagues investigated a total of 104 children with CVC placement and demonstrated that neither insertion site nor catheter type were significant risk factors for thrombosis and that acute lymphoblastic leukemia was a major clinical risk factor for thrombosis [11]. A recent study showed ultrasound guidance and site selection were potential modifiable risk factors in the development of CADVT in pediatric patients [12]. However, the reliability of the data is limited by the small sample size, few catheter-related thrombosis events, few factors, and heterogeneity in the outcome definitions [13, 14]. Furthermore, current screening guidelines for venous thromboembolism risk which are developed from incomplete pediatric data and extrapolated from adult data have low sensitivity for CADVT in hospitalized children [15]. In a preliminary experiment in this study, the traditional regression model did not work very well in these complicated situations. In a sense, the poor performance of traditional models also limits our ability to really explore the risk factors for CADVT in children.

Since risk factors for the development of CADVT are not well understood in children in intensive care setting, the purpose of this retrospective cohort study was to identify significant clinical risk factors independently associated with the development of CADVT in pediatric intensive care patients based on fusion machine learning models.

## Methods

This retrospective study was approved by the Institutional Review Board of Children’s Hospital of Zhejiang University School of Medicine with a waiver of informed consent. In this study, data were collected on pediatric patients who were admitted to the intensive care unit between December 2015 and December 2018 at the 1900-bed Children’s Hospital, Zhejiang University School of Medicine. It has 119 critical care beds in 4 intensive care units: pediatric ICU (PICU), surgical ICU (SICU), cardiac ICU (CICU) and neonatal ICU (NICU). The clinical data from a total of 11,814 patients were recorded in a public pediatric intensive care (PIC) database [16]. Every CVC line in these ICU will be assessed daily as standard of care and the assessment records were used for this study. The thrombus was confirmed with Doppler ultrasound or computed tomography and recorded in the adverse event reports. As the publicly available PIC database does not contain CVC-related records, additional daily catheter assessment records and CVC-related adverse event report data were aligned with the clinical data in PIC database. The inclusion criteria in this study required that patients received at least one CVC placement during the study period. The exclusion criteria are defined as patients with thrombosis either present before hospital admission or before CVC placement.

### Data collection and preprocessing

Patient and CVC-specific data were collected from the PIC database and CVC-related records in different clinical information systems. The primary outcome of interest in the present study was the occurrence of CADVT. The variables analyzed included age, sex, primary diagnosis, surgery before CVC insertion, intensive care unit type (PICU, SICU, CICU and NICU), CVC insertion time, CVC removal time, catheter-related characteristics (type and size), length of intensive care unit stay, length of hospital stay, and hospital mortality. The duration of CVC was calculated as the time of catheter removal or death minus the time of catheter insertion. To explore more potential risk factors, we included 7 vital signs, 49 laboratory test items, and 6 drug categories in this study. The details of these factors and the define of drug categories are shown in the supplemental material. For 56 vital signs and laboratory test items with multiple repeated measurements, statistical values such as the mean, standard deviation, minimum, median, and maximum were used for the analysis.

### Statistical analysis

All statistical analyses were performed using the published package in the Python and R programming environments. The patients were categorized according to whether they had experienced the primary outcome of CADVT. All continuous data between patients with and without CADVT are reported as the mean value ± standard deviation with median and interquartile range (IQR), and compared using the Mann-Whitney U test. All categorical data are reported as counts (percentages) and compared using the chi-square test. The odds ratio and 95% confidence interval, which indicate the relative likelihood of CADVT per additional episode of variability, were calculated by the epitools package in R (version 3.6.0). For statistical hypothesis testing purposes, we considered a *p*-value less than 0.05 to indicate significance.

### Machine learning model

The logistic regression (LR) model and two superior performance machine learning models, random forest (RF) and gradient boosting decision tree (GBDT), were used as three basic models for model fusion. Two ensemble learning strategies, i.e., stacking and blending [17], which combine multiple primary learners through secondary learners, are selected to complete the CADVT prediction task. The positive patients (with CADVT) in the data set accounted for less than 10% of the total. The phenomenon of class imbalance or class skew can cause unreasonable evaluation of the two-class classifier. The SMOTE method [18] was adopted as a processing method for unbalanced data before training. The factors without contribution (the performance did not change when the factor was removed from the input) in at least two basic models were filtered before the fusion model was trained. We used accuracy, recall, area under the ROC curve (AUC), and average precision (AP) to evaluate the performance of the prediction model. A 5-fold cross validation approach was used in the evaluation. All these were conducted under the scikit-learn Python module.

### Impact of risk factors

We used the impact of variables on the accuracy of the fusion model to determine whether it is a high-risk factor. After excluding a certain independent variable, the difference between the model accuracy and the original accuracy was calculated. In a circular manner, the influence of all factors on the model accuracy is obtained. The larger the result is, the greater the influence of this factor is on the model’s prediction accuracy. The overlapping risk factors in the two fusion approaches were reported.

## Results

Of all patients who were admitted to intensive care units between December 2015 and December 2018, 3871 children who received CVC placement among 3927 admissions and met our inclusion criteria were included in this study. The detailed patient information is shown in Table 1. The mean age of the cohort was 33.29 ± 41.52 months, with a median of 15.07 and IQR [4.96, 45.65], and 2065 (52.58%) patients were male. The spectrum of primary diagnoses included hundreds various cardiac, oncologic, infectious, gastrointestinal, and neurologic conditions. These were broadly categorized as congenital heart disease (CHD) [*n* = 1873(47.70%)], infection or inflammation [*n* = 413(10.52%)], other congenital disease [*n* = 239(6.09%)], cancer [*n* = 217(5.53%)], cysts and mass [*n* = 231(5.88%)], intracranial space-occupying lesion [*n* = 125(3.18%)], bleeding [*n* = 46(1.17%)], and other [*n* = 754(19.20%)] (detail definition of these disease groups were shown in supplemental Table S2). Of these enrolled patients, 387 (9.85%) experienced CADVT, which were confirmed by duplex ultrasound or computed tomography. The mean CVC dwell time of CADVT cases was 7.5 ± 9.8 days, with a median of 6.1 and IQR [2.9,9.4].

**Table 1:** Patient characteristics stratified by patient CADVT status

Characteristics	Patients with CADVT *n* = 387(9.85%)	Patients without CADVT *n* = 3540(90.15%)	*P*-value
Gender			**< 0.001** ^*^
Male	**242 (62.5%)**	**1823 (51.5%)**	
Female	**145 (37.5%)**	**1717 (48.5%)**	
Age (months)	**Mean 44.3 ± 48.4** **Median 21.8 [6.4,72.1]**	**Mean 32.1 ± 40.5** **Median 14.5 [4.8,43.2]**	**< 0.001** ^**#**^
Weight (kg)	**Mean 15.4 ± 12.4** **Median 11.1 [7.0,18.7]**	**Mean 12.9 ± 10.9** **Median 9.5 [6.3,15.5]**	**0.007** ^**#**^
Diagnosis			**< 0.001** ^*^
Bleeding	**10 (2.6%)**	**36 (1.0%)**	
Cancer	**19 (4.9%)**	**198 (5.6%)**	
CHD	**60 (15.5%)**	**1813 (51.2%)**	
Intracranial space-occupying lesion	**40 (10.3%)**	**85 (2.4%)**	
Cysts and mass	**13 (3.4%)**	**218 (6.2%)**	
Premature infant	**2 (0.5%)**	**27 (0.8%)**	
Infection/inflammation	**90 (23.3%)**	**323 (9.1%)**	
Other congenital disease	**15 (3.9%)**	**224 (6.3%)**	
Other	**138 (35.7%)**	**616 (17.4%)**	
Length of hospital stay (days)	**Mean 29.3 ± 28.1** **Median 21.6 [14.1,33.8]**	**Mean 18.0 ± 16.4** **Median 14.0 [9.8,21.0]**	**< 0.001** ^**#**^
Length of ICU stay (days)	**Mean 12.7 ± 18.0** **Median 7.8 [2.0,16.1]**	**Mean 4.5 ± 12.0** **Median 1.8 [0.9,4.0]**	**< 0.001** ^**#**^
ICU admission			**< 0.001** ^*^
CICU	**75 (19.4%)**	**2192 (61.9%)**	
NICU	**5 (1.3%)**	**122 (3.4%)**	
PICU	**157 (40.6%)**	**344 (9.7%)**	
SICU	**150 (38.8%)**	**882 (24.9%)**	
Catheter type			**0.027** ^*^
Single lumen	**311 (80.4%)**	**2660 (75.1%)**	
Double lumen	**76 (19.6%)**	**880 (24.9%)**	
Catheter model			**< 0.001** ^*^
18G	**94 (24.3%)**	**861 (24.3%)**	
20G	**19 (4.9%)**	**62 (1.8%)**	
22G	**217 (56.1%)**	**1803 (50.9%)**	
4.0Fr	**10 (2.6%)**	**287 (8.1%)**	
5.0Fr	**37 (9.6%)**	**493 (13.9%)**	
Other	**10 (2.6%)**	**34 (1.0%)**	
Dwell time (day)	**Mean 7.5 ± 9.8** **Median 6.1 [2.9,9.4]**	**Mean 4.2 ± 4.8** **Median 2.7 [0.9,5.9]**	**< 0.001** ^**#**^
Surgery			**< 0.001** ^*^
True	**252 (65.1%)**	**3119 (88.1%)**	
False	**135 (34.9%)**	**421 (11.9%)**	
Mortality	**20(5.2%)**	**85(2.4%)**	**0.003** ^*^

^*^ chi-square test; ^**#**^ Mann-Whitney U test

Baseline demographics, CVC-specific data, and some other clinical characteristics evaluated for 3927 patients stratified by CADVT are summarized in Table 1. Detailed information on 56 vital signs and laboratory test items is summarized in Supplemental Table S1. Patients with thrombosis were older, with a mean age of 44.3 ± 48.4 months (Median 21.8 IQR[6.4,72.1]) versus a mean age of 32.1 ± 40.5 months (Median 14.5 IQR[4.8,43.2]) for those without thrombosis. CADVT happened more frequent in boys than in girls. Compared with CHD (the reference category), the odds of experiencing CADVT were significantly higher for patients with intracranial space-occupying lesions, bleeding, and infection/inflammatory diseases as shown in Table 2. Many rare diseases were classified as “Other” which contribute more than one third of CADVT events. The patient’s ICU type, history of surgery, catheter dwell time, and types and sizes of catheters were significantly associated with the occurrence of CADVT. Furthermore, the length of intensive care unit stay and hospital stay were significantly longer for patients who experienced CADVT than for patients without CADVT. Patients with CADVT had a comparably higher mortality rate than those without CADVT (5.2% vs. 2.4%). There are many vital signs, and lab test items also show significant differences between patients with and without CADVT.

**Table 2:** CADVT odds ratios by different disease groups

Disease group	OR	95% CI lower	95% CI upper	*P* value
CHD	1	NA	NA	NA
Bleeding	8.45391	3.7944632	17.331222	< 0.001
Cancer	2.913149	1.6589146	4.899299	< 0.001
Intracranial space-occupying lesion	14.180768	8.9495734	22.367144	< 0.001
Cysts and mass	1.818107	0.9384196	3.267068	0.057
Premature infant	2.390952	0.3511494	8.276947	0.266
Infection/Inflammation	8.400088	5.9474207	11.947027	< 0.001
Other congenital disease	2.038201	1.0962857	3.562076	0.016
Other	6.753561	4.9438764	9.328297	< 0.001

**Fig. 1:**
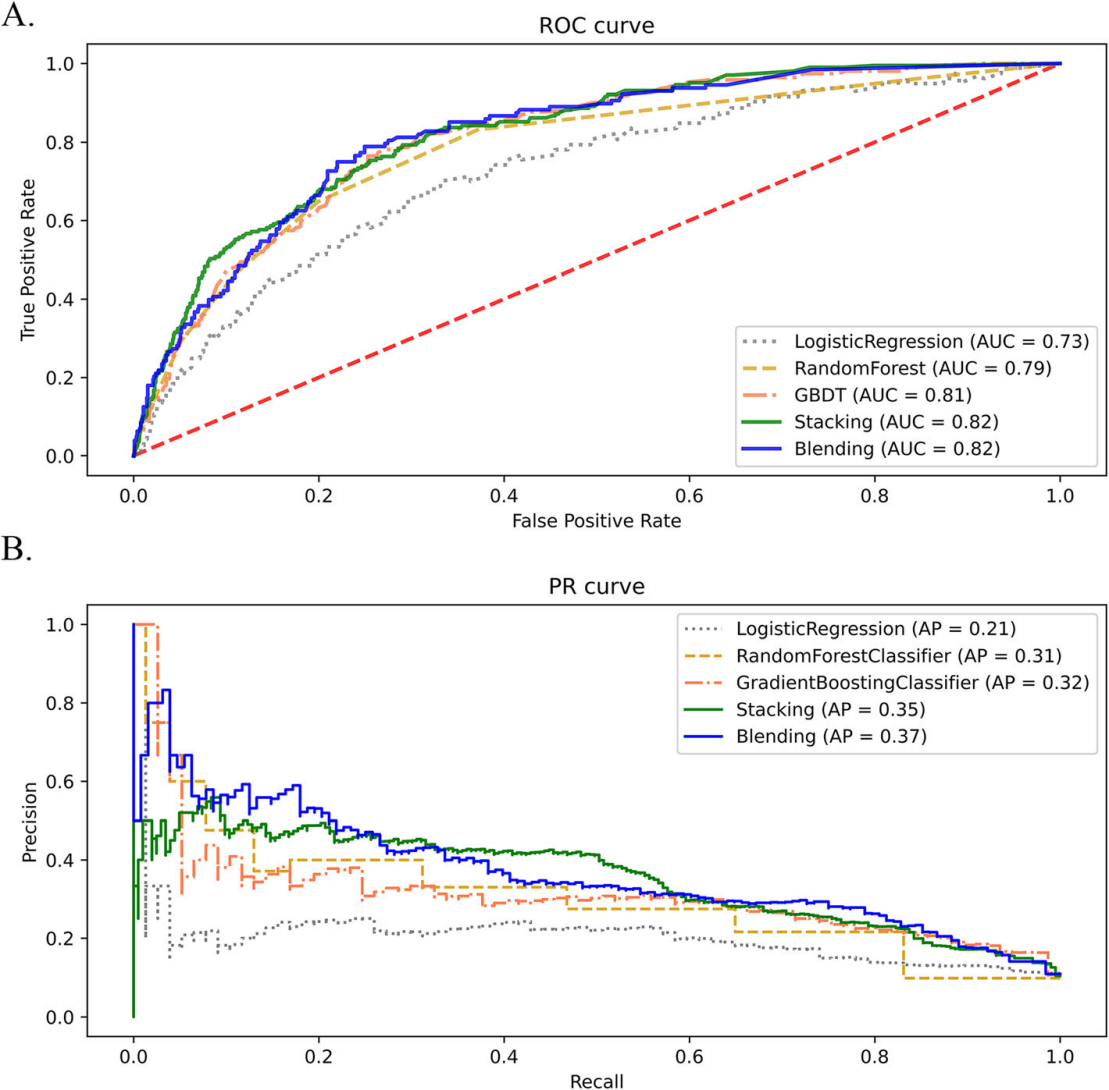
The CADVT prediction performance of 3 basic models and 2 fusion models. (A) ROC curves (B) PR curves

A total of 478 independent variables were screened and reduced to 74 based on their contributions in the three basic models. Based on the selected features, the accuracy, recall, AUC and AP of the three basic models and two fusion models are shown in Table 3. The two machine learning models RF and GBDT achieved better performance, especially the recall compared with traditional LR model. The two fusion models further increased the recall rate over 0.84 and elevated the AUC and AP as shown in Fig. 1.

**Table 3:** The performance of the three basic models and two fusion models

	accuracy	recall	AUC	AP
LR	0.76	0.53	0.73	0.21
RF	**0.77**	0.70	0.79	0.31
GBDT	0.72	0.79	0.81	0.32
Stacking	0.64	0.84	**0.82**	0.35
Blending	0.65	**0.85**	**0.82**	**0.37**

The top 20 high-impact factors in the two fusion CADVT prediction models are shown in Table 4. A total of 11 factors that overlapped in the two fusion models are asterisked in Table 4 and will be discussed separately below.

**Table 4:** The top 20 high-impact factors in the two fusion CADVT prediction models

	Stacking		Blending	
rank	Factor type	Factor	Factor type	Factor
1	Lab test	Standard base excess (SBE)*	Lab test	PH*
2	Lab test	Thrombin time (TT) difference*	Surgery	Past history*
3	Drug	Dehydrating agent*	Lab test	Oxygen partial pressure (PaO_2_)*
4	Lab test	Bicarbonate*	Lab test	calcium
5	Lab test	Platelet count	Lab test	Plasma D-dimer (DD)*
6	Lab test	Oxygen partial pressure (PaO_2_)*	Lab test	Bicarbonate*
7	Lab test	Plasma D-dimer (DD)*	Admission ICU	SICU
8	Lab test	Basophil absolute value*	Lab test	Actual alkali surplus
9	Lab test	Thrombin time (TT)*	Drug	Dehydrating agent*
10	Lab test	Carboxyhemoglobin	Drug	Anticoagulant drugs
11	Vital sign	Diastolic blood pressure	Lab test	Thrombin time (TT) difference*
12	Surgery	Past history*	Lab test	Thrombin time (TT)*
13	Lab test	Anion gap	Lab test	Mean hemoglobin concentration
14	Sex	Male	Catheter model	18G
15	Lab test	Lymphocytes (LY%)*	Lab test	Basophil absolute value*
16	Catheter type	Single lumen	Lab test	Lymphocytes (LY%)*
17	Diagnosis	Cysts and mass	Catheter type	Double lumen
18	Lab test	Normal control prothrombin time	diagnosis	Cancer
19	Vital sign	Blood oxygen saturation	Lab test	Standard base excess (SBE)*
20	Lab test	PH*	Lab test	Neutrophil absolute value

* Indicates the overlap in the two fusion models

### Acid-base balance related factors

Acid-base balance-related factors, such as pH, standard base excess (SBE), oxygen partial pressure (PaO_2_), and bicarbonate, were identified as risk factors in both fusion models. First, the pH value was relatively higher in patients with CADVT (7.42 ± 0.08 vs. 7.40 ± 0.07, *p* < 0.001). The reason for this finding may be that the bicarbonate (a base) in the blood is higher in patients with CADVT (27.4 ± 5.3) than in patients without CADVT (25.4 ± 4.7). The SBE was also higher in patients with CADVT (3.1 ± 5.6) than in patients without CADVT (1.0 ± 5.1). The PaO_2_ was lower in patients with CADVT (131.8 ± 58.1 vs. 145.3 ± 68.5, *p* < 0.001). All these factors contribute to the increase in pH value. Based on increased PaCO2 in CADVT patients shown in supplemental Table S1, it is more like a metabolic acid-base disturbances.

### Coagulation-related factors

Coagulation-related factors, such as thrombin time (TT) and TT difference (with reference) and plasma D-dimer (DD), were identified in both models. The DD increased in patients with CADVT (3.4 ± 4.9 vs. 2.2 ± 3.8, *p* < 0.001). A prolonged and more volatile TT (23.3 ± 9.1 vs. 20.8 ± 6.5, *p* < 0.001) and TT difference (4.1 ± 9.1 vs. 1.7 ± 6.5, *p* < 0.001) were observed in patients with CADVT.

### Drug-related factors

The dehydrating agents which also called as diuretics were identified as a high-impact factor in both fusion models. Although the anticoagulant drug was identified in the blending model, it was not shown in the top 20 factors in the stacking model.

### Blood cell-related factors

Two types of white blood cells, basophils and lymphocytes, were identified by both models. Lymphocytes (LY%) were lower in patients with CADVT (26.3 ± 16.7 vs. 31.3 ± 18.4, *p* < 0.001), and the basophil absolute value was also lower in patients with CADVT (0.0268 ± 0.0329 vs. 0.0307 ± 0.0354, *p* < 0.001).

### Surgery related factors

The CADVT rate was 24.28% in patients without surgery in ICU. At the same time, it is about 7.48% of patients experienced surgery.

## Discussion

In this retrospective analysis, we sought to identify clinical factors associated with CADVT in pediatric intensive care patients using a fusion machine learning model with good prediction performance. Traditional logistic regression analyses were usually used to identify risk factors for VTE [19]. In this study, the logistic regression model was not quite satisfactory because of the relatively low recall rate (0.53) which means about 47% CADVT will not be correctly predicted. In a task that predicts events with low incidence, we care more about the recall rate of the model and do not want to miss any events. We introduced two more modern machine learning models (RF and GBDT) with better performance, especially providing a higher recall rate. Furthermore, two ensemble learning approaches that make predictions by using a meta-model trained from a pool of base models achieved much better performance, especially the recall rate. An AUC of 0.82 is quite satisfactory in such a complicated prediction task. These fusion models have the potential to be used as a decision support tool for thromboprophylaxis.

Although many factors were significantly different between patients with and without CADVT, only 11 factors were identified independently and highly contributed to both fusion models. Due to the fusion AI model is less interpretable, we will try to explain these factors and their potential mechanisms associated with CADVT based on the literature separately in below.

Although there are no reports on the relationship between CADVT and the blood acid-base balance, some studies have proposed a mechanism by which the procoagulant properties of blood are impaired at subnormal pH values in trauma [20, 21]. These studies showed that reduced blood pH causes bleeding. It is reasonable to infer that a relatively high pH may be a risk factor for blood coagulation. This finding provides a strategy for thromboprophylaxis that monitors the pH level and avoids even a small increase in pH. Due to the statistically small changes in pH between the two groups of patients, there is a need for further research on how to set individual pH change monitoring thresholds in the clinic. Some studies have also reported that thrombus formation is increased under conditions of hypoxia [22]. At the same time, some data also showed that severe hyperoxia events appeared to be associated with increased mortality in PICU [23]. Therefore, monitoring the pH and controlling PaO_2_ is an operable option for thromboprophylaxis in pediatric intensive care units.

An interesting finding is coagulation-related factors. Although many of the patients with CVC had prolonged TT due to anticoagulation prophylaxis, the patients with CADVT had an unexpectedly longer TT and larger variation (23.3 ± 9.1 vs. 20.8 ± 6.5). TT alone does not seem to be the determining factor. Several studies have reported that DD increased significantly in DVT [25, 25] and was strongly and positively related to the occurrence of future venous thrombosis [26, 29]. We confirmed that DD is also a potential predictor of CADVT in pediatric intensive care settings. However, it is unclear if the elevated of DD causes clot or is the result of clot. In any case, monitoring DD facilitates the timely detection of CADVT.

A total of 6 CADVT-related drug categories were included in this study. Only the dehydration agent, such as mannitol, glycerol fructose, and furosemide etc., that is widely used to reduce brain swelling and intracranial pressure appears in the final list. The negative finding of anticoagulant drugs, procoagulant drugs, vasoconstrictive drugs, vasodilators, and sedative drugs indicates that the current clinical practice for thromboprophylaxis and anticoagulation management is very common and effective. The association of thrombosis and dehydration has been reported by many studies [28]. The dehydration of infants seems more sensitive to thrombosis [29–31]. This finding was also supported by the high odds ratios for CADVT in patients with intracranial space-occupying lesions and bleeding, such as cerebral hemorrhage. This result reminds clinicians to pay more attention to patients who receive dehydrating agents during CVC dwell time.

Based on recent studies, there is a significant correlation between the neutrophil to lymphocyte ratio (NLR) and platelet to lymphocyte ratio (PLR) and the incidence of DVT [32, 33]. In this study, we also identified lower LY% as a potential predictor. Neutrophils (NE%) were also higher in the CADVT cohort (63.9 ± 18.5 vs. 58.7 ± 20.4). If we estimated the NLR using the mean value of NE% and LY% in the two groups, the NLR also increased in the CADVT cohort (2.4296 vs. 1.8754). The platelet count is only identified as a high impact factor in one of the fusion models. The PLR and NLR are also well-known inflammatory markers that reflect the activity of many inflammatory diseases [34]. Inflammatory diseases with higher ORs in CADVT were also confirmed in this study as shown in Table 2. It is necessary to delve into the potential association between these two indicators and CADVT in pediatric intensive care units.

**Fig. 2:**
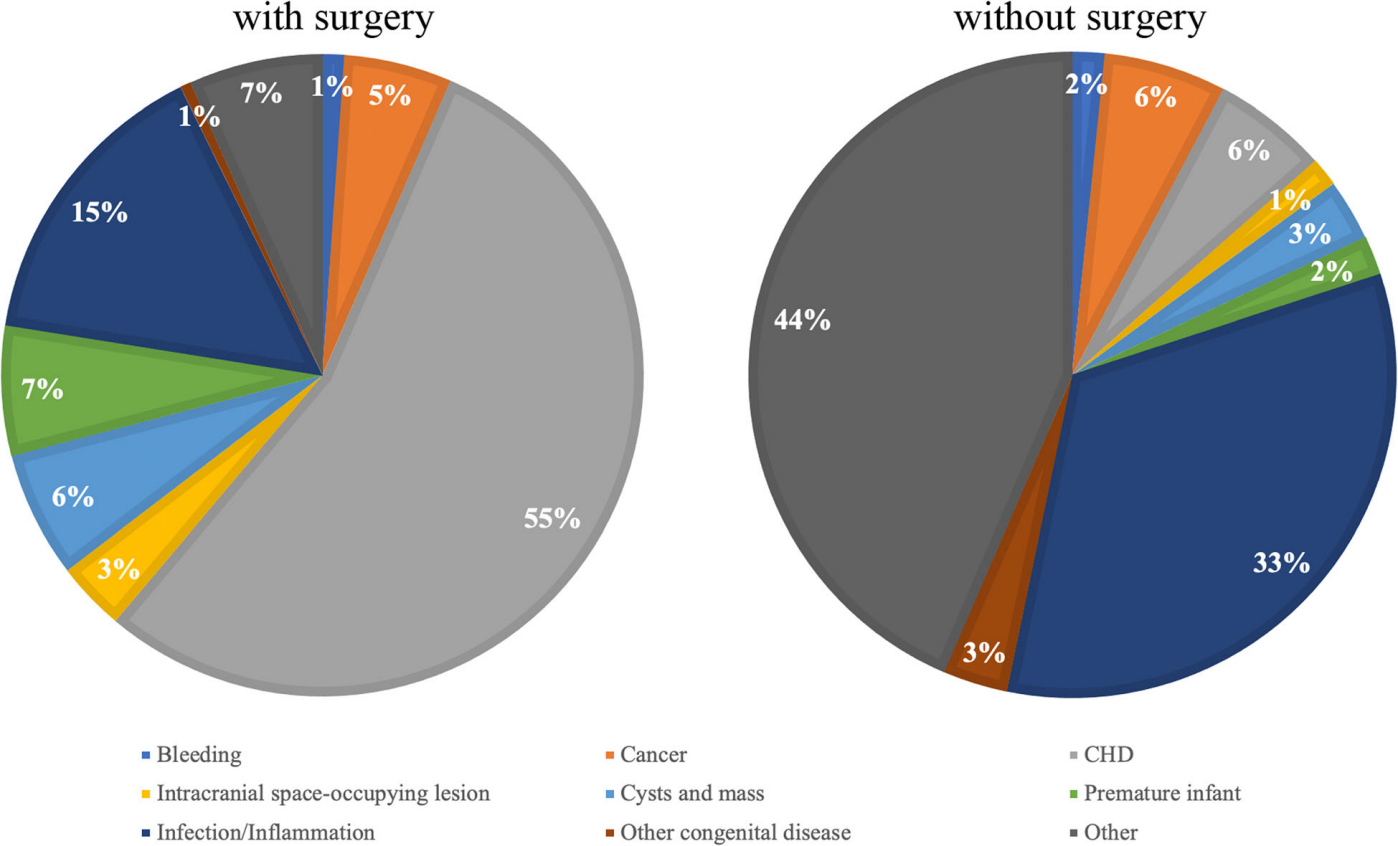
The differences in disease in patients with and without a surgical history

The other important factor identified was surgery history. Patients who experienced surgery, especially the CHD procedures, had a lower risk of CADVT compared to those who have not experienced surgery. One possible explanation is the difference in the distribution of disease between patients who had surgery and patients who did not have surgery as shown in Fig. 2. Because thrombosis is one of the most common complications affecting children with CHD, thromboprophylaxis and anticoagulation management are routinely performed in the perioperative period of CHD. More than half of surgeries were received by patient with CHD. And they also received thromboprophylaxis which contribute to the lower risk of patient experienced surgery. In patients without a surgical history, in addition to a smaller proportion of CHD, there was a significant increase in the proportion of rare diseases that were not clearly classified. This result suggests that we need to take a closer look at the association of these uncommon diseases with CADVT in the future. Given that the disease groups used in the model were expert-defined groups, we further calculated the risk of CADVT for specific disease subgroups according to standard ICD-10 disease classifications as shown in Table 5. The result further confirmed that congenital malformation disorders with predominantly CHD have a lower risk of CADVT under current thromboprophylaxis, while the risk of CADVT was significantly increased in some traumatic conditions especially those causing central nervous system injury.

**Table 5:** The CADVT odds ratios of different disease groups based on ICD-10 classification

ICD10	Count	OR	*P* value
A00-B99 Certain infectious and parasitic diseases	33	**4.056**	**< 0.001**
C00-D48 Neoplasms	216	0.876	0.724
D50-D89 Diseases of the blood and blood-forming organs and disorders involving the immune mechanism	13	0.761	1.0
E00-E90 Endocrine, nutritional and metabolic diseases	27	2.093	0.180
F00-F90 Mental and behavioural disorders	1		
G00-G99 Diseases of the nervous system	139	**4.007**	**< 0.001**
H00-H59 Diseases of the eye and adnexa	2	9.168	0.187
H60-H95 Diseases of the ear and mastoid process	1		
I00-I99 Diseases of the circulatory system	366	1.032	0.854
J00-J99 Diseases of the respiratory system	326	**2.152**	**< 0.001**
K00-K93 Diseases of the digestive system	282	1.186	0.406
L00-L99 Diseases of the skin and subcutaneous tissue	2		
M00-M99 Diseases of the musculoskeletal system and connective tissue	7	**6.906**	**0.025**
N00-N99 Diseases of the genitourinary system	7		
O00-O99 Pregnancy, childbirth and the puerperium	0		
P00-P96 Certain conditions originating in the perinatal period	90	0.648	0.373
Q00-Q99 Congenital malformations, deformations and chromosomal abnormalities	1952	**0.173**	**< 0.001**
R00-R99 Symptoms, signs and abnormal clinical and laboratory findings, not elsewhere classified	316	**3.291**	**< 0.001**
S00-T98 Injury, poisoning and certain other consequences of external causes	102	**3.106**	**< 0.001**
V01-Y98 External causes of morbidity and mortality	23	**5.997**	**< 0.001**
Z00-Z99 Factors influencing health status and contact with health services	22	0.434	0.718

In addition to the 11 high impact factors, there are also some other interesting features that need to be discussed here. In contrast to previous findings that sex was not a significant risk factor for CVC-related thrombosis [8, 35], there was an increased thrombotic risk when the patient’s sex was male (OR 1.57, 95% CI 1.27–1.95, *P* < 0.001) in this dataset. One of the potential explanations is boy-girl ratio different in injuries such as traumatic brain injury which is an important source of CADVT in this center [36]. CVC characteristics, including the number of lumens, size of catheter, and catheter dwell time, were evaluated in this study. The double lumen CVC seems safer in this study. However, we did not find that the size of catheters had an independent association with CVC-related thrombosis. Several previous studies demonstrated an increased risk of thrombosis as catheter diameter increased, but this factor has not been consistently demonstrated across all studies [35, 37]. Catheter dwell time has also been suggested as a risk factor for catheter-related complications, with some studies finding longer dwell times associated with complications [38] and others finding shorter dwell times associated with complications [39]. Our study found that a longer dwell time was associated with a higher risk of CVC-related thrombosis. These inconsistent findings may be explained by the different mechanisms of thrombus formation: cases with severe vascular endothelial injury may have CVC-related thrombosis in the early stage, while thrombosis may occur slowly because of slow blood flow and local blood flow morphological changes. However, given the complex association of catheter dwell time with CADVT, further analysis is needed.

There are several limitations of this retrospective study. First, this is a retrospective study at a single center, and the results may be subject to bias or incomplete information; thus, larger multicenter cohort studies will be needed in the future. Second, potential confounding variables, such as the location of catheter insertion and indication for CVC insertion, were not evaluated. Third, the disease group schema used in this study is not strictly defined in the grouping based on their Chinese terms, and findings during the study were also used to separate special diseases, such as “intracranial space occupying lesions” with higher risk of CADVT were defined as a separated disease group and it may also belong to cancers in some cases. In addition, only the primary discharge diagnosis was used to label patient. It should include more diseases information in the future study. Nonetheless, to our knowledge, this is the largest study to date to identify clinical risk factors associated with CADVT in pediatric intensive care patients when compared with other analyses [8, 11, 12]. Furthermore, we introduced a better performing fusion model to identify high-impact factors. However, some identfied risk factors differed only slightly between the two groups failing to reach clinical significance or even statistical significance. Whether there is a potential bias in this approach has not been studied in depth.

## Conclusions

In conclusion, children in intensive care are at high risk for CADVT, which occurs in approximately 10% of these patients. Two fusion models based on 3 basic models were developed and achieved quite satisfactory performance in CADVT prediction. From 478 variables, 11 independent and high-impact CADVT risk factors were identified based on the fusion model. These findings provide potential biomarkers and measures for thromboprophylaxis in pediatric intensive care settings.

## Supplementary information

**Additional file 1.**

## Data Availability

The datasets used and/or analyzed during the current study are available from the corresponding author on reasonable request.

## Abbreviations

CVC: Central venous catheter
CADVT: Catheter-associated deep venous thrombosis
CRT: Catheter-related thrombosis
DVT: Deep venous thrombosis
PIC: Pediatric intensive care database
LR: Logistic regression
RF: Random forest
GBDT: Gradient boosting decision tree
ROC: Receiver operating characteristic curve
AUC: Area under the ROC curve
AP: Average precision
SBE: Standard base excess
PaO_2_: Oxygen partial pressure
TT: Thrombin time
DD: D-dimer
LY%: Lymphocyte%
NLR: Neutrophil to Lymphocyte Ratio
PLR: Platelet to Lymphocyte Ratio
NE%: Neutrophil%
OR: Odds ratio
CI: Confidence interval
