# Supplementary material for: Risk factors for central venous catheter-associated deep venous thrombosis in pediatric critical care settings identified by fusion model
Source: Thromb J. 2022 Apr 12;20:18. doi: 10.1186/s12959-022-00378-y (PMC9004113; doi:10.1186/s12959-022-00378-y)
Supplement: Supplementary file 1 — Additional file 1. [file 12959_2022_378_MOESM1_ESM.docx]

**Risk Factors for Central Venous Catheter-Associated Deep Venous Thrombosis in Pediatric Critical Care Settings Identified by Fusion Model**

Haomin Li ^a,#^, PhD, Yang Lu^a,b^, MS, Xian Zeng^a,b^, BS, Yuqing Feng^a^, MS, Cangcang Fu^a^ ,MSN, Huilong Duan^b^, PhD, Qiang Shu^a^,MD, Jihua Zhu^a,#^, BN

Supplementary Material

Factors in this study

(1) The patient's age, gender, weight, and whether or not the operation was performed;

(2) Central venous catheter type (single lumen / double lumen); catheter model (18G / 20G / 22G / 4.0Fr / 5.0Fr / other);

(3) Admission ICU (CICU / NICU / PICU / SICU);

(4) Disease diagnosis results（bleeding / cancer / CHD (congenital heart disease) / intracranial space-occupying lesion / cysts and mass / premature infant / systemic infection / other congenital disease / other）.

(5) 7 vital signs (body temperature / pulse / heart rate / respiration / oxygen saturation / systolic blood pressure / diastolic blood pressure);

(6) 49 laboratory test items (Subject active partial thromboplastin time / plasma D-dimer / fibrinogen / international standardized ratio / normal control active partial thromboplastin time / normal control prothrombin time / normal control thrombin time / be Examiner's prothrombin time / subject's thrombin time / eosinophils / neutrophil absolute value / neutrophils / hematocrit / hemoglobin / lymphocyte absolute value / lymphocyte / average hemoglobin amount / average Hemoglobin concentration / average red blood cell volume / average platelet volume / platelet packed volume / platelet distribution width / platelet count / red blood cell count / red blood cell distribution width / white blood cell count / absolute value of basophils / absolute value of monocytes / monocytes / Eosinophil absolute value / calcium / chlorine / potassium / sodium / bicarbonate / methemoglobin / carbon dioxide partial pressure / pH / oxygen partial pressure / Standard base excess (SBE) / oxygen saturation / actual base residual / anion gap / carboxyhemoglobin / Glucose (electrode method) / Lactate / Thrombin time difference / Prothrombin time difference / Activated partial thromboplastin time difference).

(7) 6 drug therapy categories

(7.1) Anticoagulant drugs (heparin sodium injection / nadroparin calcium injection / low molecular weight heparin calcium injection / warfarin sodium tablets / aspirin enteric-coated tablets / dipyridamole tablets / clopidogrel bisulfate tablets);

(7.2) Procoagulant drugs (Hemocoagulase for injection / vitamin K1 injection / human coagulation factor Ⅷ / recombinant human coagulation factor Ⅷ for injection / recombinant human coagulation factor Ⅷa for injection / human prothrombin complex / human fiber Protein / Tranexamic acid injection / Tranexamic acid for injection / Tranexamic acid injection / Sulfenac injection / Poster pituitary injection);

(7.3) Dehydrating agent (20% mannitol injection / glycerol fructose sodium chloride injection / glycerol fructose injection / furosemide tablets / furosemide injection / 20% human albumin injection / concentrated sodium chloride Injection / 10% sodium chloride injection);

(7.4) Vasoconstrictive drugs (epinephrine hydrochloride injection / norepinephrine bitartrate injection / isoproterenol hydrochloride injection / phenylephrine hydrochloride injection);

(7.5) Vasodilators (dopamine hydrochloride injection / phentolamine mesylate for injection / phentolamine mesylate injection / nitroglycerin injection);

(7.6) Sedative drugs (diazepam injection / diazepam tablets / estazolam tablets / midazolam injection / clonazepam tablets / phenobarbital sodium for injection / phenobarbital tablets / Phenobarbital Sodium Injection).

**Table S1 Vital signs and lab tests stratified by patient CADVT status**

| **Item** | | **Patients with CADVT** | **Patients without CADVT** | **P-value** |
| --- | --- | --- | --- | --- |
| Vital sign item | body temperature | 37.1±3.1 | 37.1±13.4 | 0.626 |
|  | pulse | 128.7±28.5 | 131.9±26.9 | <0.001 |
|  | Heart rate | 122.3±30 | 127.2±27.6 | <0.001 |
|  | Breathing rate | 35.2±29.1 | 36.0±71.7 | <0.001 |
|  | Blood oxygen saturation | 97.7±7.3 | 97.5±7.3 | <0.001 |
|  | Diastolic blood pressure | 56.2±16.4 | 54.5±15.4 | <0.001 |
|  | Systolic blood pressure | 97.5±19.3 | 96.7±18.8 | 0.001 |
| Lab test item | Subject activated partial thromboplastin time | 42.6±26 | 39.3±19.1 | <0.001 |
|  | Plasma D-dimer (DD) | 3.4±4.9 | 2.2±3.8 | <0.001 |
|  | Fibrinogen | 2.3±1.3 | 2.3±1.1 | 0.411 |
|  | International normalized ratio | 1.2±0.5 | 1.2±0.4 | 0.088 |
|  | Normal control activated partial thromboplastin time | 27.9±1.7 | 27.6±1.7 | <0.001 |
|  | Normal control prothrombin time | 11.8±0.3 | 11.8±0.5 | 0.022 |
|  | Normal control thrombin time | 19.1±0.6 | 19.1±0.6 | <0.001 |
|  | Prothrombin time (PT) | 13.8±5.8 | 14±5.2 | 0.07 |
|  | Thrombin time (TT) | 23.3±9.1 | 20.8±6.5 | <0.001 |
|  | Eosinophils | 0.9±1.7 | 1.1±2 | <0.001 |
|  | Neutrophil absolute value | 7.6±5.8 | 7.0±5.2 | <0.001 |
|  | Neutrophils (NE%) | 63.9±18.5 | 58.7±20.4 | <0.001 |
|  | Hematocrit | 32.4±5.9 | 33.1±6.2 | <0.001 |
|  | Hemoglobin | 104.7±19.4 | 107.5±20.8 | <0.001 |
|  | Absolute lymphocyte value | 2.5±1.8 | 3.1±2.2 | <0.001 |
|  | Lymphocyte (LY%) | 26.3±16.7 | 31.3±18.4 | <0.001 |
|  | Average hemoglobin | 28.3±2.5 | 28.4±2.6 | <0.001 |
|  | Mean hemoglobin concentration | 323.5±15.3 | 328.4±13.6 | <0.001 |
|  | Mean red blood cell volume | 87.4±7 | 86.6±7.1 | <0.001 |
|  | Mean platelet volume | 9.7±1.3 | 9.6±1.3 | <0.001 |
|  | Platelet packed volume | 0.3±0.1 | 0.3±0.1 | 0.951 |
|  | Platelet distribution width | 14.5±2.7 | 14.3±2.9 | <0.001 |
|  | Platelet count | 303.1±172.3 | 307.8±169.1 | 0.036 |
|  | Red blood cell count | 3.7±0.7 | 4.2±43.4 | 0.056 |
|  | Red blood cell distribution width | 15.5±2.9 | 14.7±2.5 | <0.001 |
|  | White blood cell count | 11.1±6.9 | 11.3±6 | 0.17 |
|  | Basophil absolute value | 0.0268±0.0329 | 0.0307±0.0354 | <0.001 |
|  | Absolute value of monocytes | 0.9±0.7 | 0.9±0.6 | 0.716 |
|  | Monocyte | 8.1±4.2 | 7.8±4.2 | <0.001 |
|  | Eosinophil absolute value | 0.1±0.2 | 0.2±0.3 | <0.001 |
|  | calcium | 1.2±0.1 | 1.2±0.2 | <0.001 |
|  | chlorine | 106.8±8.7 | 108.4±356.9 | 0.25 |
|  | Potassium | 3.6±0.7 | 3.7±0.7 | <0.001 |
|  | sodium | 138.2±8.2 | 137.4±6.2 | <0.001 |
|  | Bicarbonate | 27.4±5.3 | 25.4±4.7 | <0.001 |
|  | Methemoglobin | 0.9±0.3 | 0.9±0.3 | <0.001 |
|  | Partial pressure of carbon dioxide (PaCO_2_) | 43.7±12.9 | 41.2±10.6 | <0.001 |
|  | pH | 7.42±0.08 | 7.40±0.07 | <0.001 |
|  | Oxygen partial pressure (PaO_2_) | 131.8±58.1 | 145.3±68.5 | <0.001 |
|  | Standard base excess (SBE) | 3.1±5.6 | 1.0±5.1 | <0.001 |
|  | Oxygen saturation | 95.3±9.7 | 95.6±9.8 | <0.001 |
|  | Actual alkali surplus | 3.0±5.2 | 0.9±4.9 | <0.001 |
|  | Anion gap | 3.9±5.7 | 5.4±5.9 | <0.001 |
|  | Carboxyhemoglobin | 1.1±0.6 | 1.1±0.5 | <0.001 |
|  | Glucose (electrode method) | 7.1±2.9 | 9.3±3.3 | 0.221 |
|  | Lactic acid | 1.7±1.7 | 1.8±1.8 | <0.001 |
|  | Thrombin time (TT) difference | 4.1±9.1 | 1.7±6.5 | <0.001 |
|  | Prothrombin time (PT) difference | 2.0±5.8 | 2.2±5.2 | 0.05 |
|  | Time difference in activated partial thromboplastin | 14.7±25.9 | 11.7±19.3 | <0.001 |

**Table S2 The detail information of disease group**

| **Diseases group** | **Disease** |
| --- | --- |
| Bleeding | Subdural hemorrhage |
|  | Cerebral hemorrhage |
|  | Intracranial hemorrhage (unknown cause) |
|  | Traumatic epidural hemorrhage |
|  | Gastrointestinal hemorrhage |
|  | Non-traumatic subdural hemorrhage |
|  | Traumatic subarachnoid hemorrhage |
|  | Pulmonary hemorrhage |
|  | Hemorrhagic shock |
|  | Traumatic subdural hemorrhage |
|  | Brainstem hemorrhage |
| Cancer | Medullary junction malignant tumor |
|  | Mediastinal tumor |
|  | Spongiform hemangioma |
|  | Mediastinal malignant tumor |
|  | Adrenal tumor |
|  | Craniopharyngioma |
|  | Angiosarcoma |
|  | Endodermal sinus tumor |
|  | Intracranial tumors of undetermined nature |
|  | Retroperitoneal tumor |
|  | Renoblastoma |
|  | Angiogenic cell tumor |
|  | Tumor of undetermined temporal lobe dynamics |
|  | Neuroblastoma |
|  | Bronchial dynamic undetermined tumor |
|  | Choroid plexus papilloma |
|  | Malignant tumor of the stomach |
|  | Aneurysm |
|  | Ovarian tumor |
|  | Sarcoma |
|  | Cerebrovascular junction tumor |
|  | Ventricular meningioma |
|  | Brain Tumor |
|  | Hepatoblastoma |
|  | Burkitt's lymphoma |
|  | Liver Tumor |
|  | Lymphangioleioma |
|  | Teratoma |
|  | Parenchymal pseudopapillary tumor |
|  | Malignant tumor of the pterygoid saddle area |
|  | Metastatic endodermal sinus tumor |
|  | Tumor |
|  | Mediastinal dynamic unspecified tumor |
|  | Renal tumor |
|  | Coronary artery tumor |
|  | Renal malignant tumor |
|  | Benign tumor of the fourth ventricle |
|  | Temporal lobe tumor |
|  | Ovarian malignant tumor |
|  | Pancreaticoblastoma |
|  | Retroperitoneal malignant tumor |
|  | Benign tumor of tongue |
|  | Neurofibromatosis |
|  | Cystadenoma |
|  | Adrenal malignant tumor |
|  | Subventricular giant cell astrocytoma |
|  | Pelvic tumor |
|  | Thymus tumor |
|  | Transverse myxosarcoma |
|  | Rhabdomyosarcoma |
|  | Malignant lymphoma |
|  | Malignant nerve sheath tumor |
|  | Aortic sinus aneurysm |
|  | Thoracic tumor |
| CHD | Ventricular septal defect |
|  | Heart disease |
|  | Atrial septal defect |
|  | Congenital heart disease |
|  | Tetralogy of Fallot |
|  | Aortic arch stenosis |
|  | Arterial catheterization |
|  | Triatrial heart |
|  | Congenital atrial septal defect |
|  | Complete ectopic pulmonary vein drainage |
|  | complete transposition of the great arteries |
|  | Atrial septal defect |
|  | congenital transposition of the great vessels |
|  | congenital right-sided aorta |
|  | Congenital double aortic arch |
| Intracranial occupying lesions | Intracranial occupying lesions |
| Cysts and mass | Cerebral cysts |
|  | Congenital cerebral cyst |
|  | Congenital Pulmonary Cyst |
|  | Common bile duct cyst |
|  | Epidural cyst |
|  | Pulmonary cyst |
|  | Retroperitoneal mass |
|  | Abdominal mass |
|  | [Brain] Arachnoid cyst |
|  | Saddle mass |
|  | Buccal mass |
|  | Pancreatic mass |
|  | Pelvic mass |
|  | Mediastinal cyst |
|  | Sacrococcygeal mass |
|  | Bronchial cyst |
|  | Brain abscess |
|  | Pulmonary mass |
|  | Brain swelling |
|  | Splenic cyst |
|  | Bile duct cyst |
|  | Congenital common bile duct cyst |
|  | Gluteal swelling |
|  | Pineal region swelling |
|  | Ovarian inclusion cyst |
|  | Cervical swelling |
|  | Rib swelling |
|  | Splenic swelling |
|  | Chest wall mass |
|  | Mesenteric cyst |
|  | Liver mass |
|  | Congenital third ventricular cyst |
|  | Benign mass of cerebral bridge |
| Other congenital disease | Congenital spinal cord embolism syndrome |
|  | Congenital cystic lung |
|  | Funnel chest |
|  | Congenital megacolon like origin disease |
|  | megacolon |
|  | Post-operative megacolon enterostomy |
|  | Congenital anal anomalies |
|  | Personal history of congenital malformations, deformities and Chromosomal abnormalities |
|  | Congenital atresia of the bile duct |
|  | Congenital ileal atresia |
|  | Congenital umbilical hernia |
|  | Congenital diaphragmatic hernia |
|  | Congenital arteriovenous fistula of the trunk |
|  | Congenital spinal cord bulge |
|  | Congenital tricuspid valve subluxation malformation |
|  | Congenital tricuspid valve insufficiency |
|  | Congenital esophageal atresia |
|  | Congenital small bowel atresia |
|  | Congenital jejunal atresia |
|  | Congenital malformation of the upper gastrointestinal tract |
|  | Congenital chondrodysplasia of the larynx |
|  | Congenital laryngeal stridor |
|  | Congenital muscular defect of the stomach wall |
|  | Congenital anal agenesis, atresia and stenosis with fistula |
|  | Congenital hypertrophic pyloric stenosis |
|  | Congenital cranial agenesis |
|  | Congenital umbilical malformation |
| Premature | Premature infants |
|  | Retinopathy of prematurity |
| Infection or inflammation | Enteritis |
|  | Bronchopneumonia |
|  | Intrahepatic cholangitis |
|  | Pneumonia |
|  | Neonatal necrotizing small bowel colitis |
|  | Sepsis |
|  | Hepatitis |
|  | Neonatal pneumonia |
|  | Acute pancreatitis |
|  | Meningoencephalitis |
|  | Infective endocardial inflammatory bullae |
|  | Ulcerative colitis |
|  | Acute septic meningitis |
|  | Parasitic infection |
|  | Non-infectious multi-organ dysfunction syndrome (MODS) |
|  | Acute upper respiratory tract infection |
|  | Acute gangrenous appendicitis |
|  | Neonatal sepsis |
|  | Post-surgical pancreatitis |
|  | Biliary ductitis |
|  | Peritonitis |
|  | Staphylococcal pneumonia |
|  | Common bile duct stones with cholecystitis |
|  | Specific acute myocarditis |
|  | Constrictive pericarditis |
|  | Migratory pneumonia |
|  | Cholecystitis |
|  | Post-infectious cough |
|  | Cellulitis |
|  | Focal encephalitis |
|  | Common bile duct stones with chronic cholecystitis |
|  | Chronic fibrous pancreatitis |
|  | Infected bursitis of the thigh |
|  | Septic meningoencephalitis |
|  | Parapharyngeal space infection |
|  | Tonsil stump infection |
|  | Myocarditis |
|  | Viral encephalitis |
|  | Enterovirus infection |
|  | Disseminated encephalitis |
|  | Aspiration pneumonia |
|  | Neonatal aspiration pneumonia |
|  | Acute laryngitis |
|  | Acute pyogenic appendicitis |
|  | Urinary tract infection |
|  | Myositis |
|  | peri-splenitis |
|  | Endocarditis with aortic atresia insufficiency |
|  | Brainstem encephalitis |
|  | Adenovirus pneumonia |
|  | Choledocholithiasis with cholangitis |
|  | Pyogenic myelitis |
|  | EBV infection |
|  | Limited encephalitis |
| Other | Convulsions |
|  | Head trauma |
|  | Foreign body in the respiratory tract |
|  | Isolated lung |
|  | Hydrocephalus |
|  | Burn (scalding) injury |
|  | Inherited metabolic diseases |
|  | Pulmonary valve atresia |
|  | Adult respiratory distress syndrome |
|  | Malnutrition |
|  | Premature closure of cranial suture |
|  | 17α-hydroxylase deficiency |
|  | Subdural effusion |
|  | Fever to be investigated |
|  | Post-operative digestive system disorders |
|  | cerebrovascular specific malformation |
|  | Severe malnutrition |
|  | Pulmonary artery atresia |
|  | Skull defect repair |
|  | Bone fracture |
|  | Skull fracture |
|  | Smoker's disease |
|  | malrotation of the bowel |
|  | Abdominal pain pending investigation |
|  | Abnormal origin of coronary arteries |
|  | intestinal adhesions |
|  | Spina bifida |
|  | Encephalopathy |
|  | mediastinal hernia |
|  | Pulmonary valve stenosis |
|  | Adrenal cortical insufficiency |
|  | Complete atrioventricular septal defect |
|  | Pulmonary emphysema |
|  | Under examination and observation after traffic accident |
|  | Strangulated bowel necrosis |
|  | Arnold Chiari malformation (A-K syndrome) |
|  | Inguinal hernia |
|  | Perforation of the digestive tract |
|  | Jaundice |
|  | Disorders of electrolyte metabolism |
|  | Intestinal obstruction |
|  | Abdominal swelling |
|  | Pulmonary artery stenosis |
|  | Paroxysmal epilepsy |
|  | Esophageal atresia with tracheoesophageal fistula |
|  | Single atrium |
|  | Traumatic brain herniation |
|  | Respiratory distress |
|  | Drowning |
|  | Cardiomyopathy |
|  | Intussusception |
|  | Diaphragmatic hernia |
|  | Non-traumatic epidural hematoma |
|  | Asphyxia |
|  | Occipital foramen magnum hernia |
|  | Common bile duct dilatation |
|  | Respiratory failure |
|  | Myelopathy |
|  | Partial pulmonary vein ectopic drainage |
|  | Diaphragmatic elevation |
|  | Specific surgical follow-up medical treatment |
|  | Pericardial effusion |
|  | Aortic constriction |
|  | Esophageal compression |
|  | Neonatal vomiting |
|  | Vitamin K deficiency |
|  | Bile duct stenosis |
|  | Williams Syndrome |
|  | Extrusion syndrome |
|  | Spinal Cord Occupational Lesions |
|  | Diabetes mellitus |
|  | Splenic Injury |
|  | Renal Failure |
|  | Hepatobiliary duct dilatation |
|  | Pulmonary hypertension |
|  | Brain contusion |
|  | Mitochondrial encephalomyopathy |
|  | Interstitial lung disease |
|  | Liver Failure |
|  | Venous sclerosis |
|  | muffled fever syndrome |
|  | Motor vehicle collision with indeterminate intent |
|  | Cardiac arrhythmia |
|  | Successful resuscitation from cardiac arrest |
|  | Mesenteric dysplasia |
|  | Wavy diaphragm |
|  | Nausea and vomiting |
|  | Abnormal liver function |
|  | Influenza |
|  | Accidental poisoning by drug overdose |
|  | Occupational liver lesions |
|  | Routine well-child examination |
|  | Multiple burns with at least one third degree burn |
|  | Bilateral sensorineural deafness |
|  | Scalp laceration |
|  | Left coronary origin pulmonary artery |
|  | Skin contusion |
|  | Pulmonary artery sling |
|  | Chronic left heart insufficiency |
|  | Chicken chest |
|  | Fall, jumping or being pushed from a height with uncertain intent |
|  | Fall or fall from a house or building structure, fall from a building |
|  | Pneumothorax |
|  | Central hypoventilation |
|  | Persistent epilepsy |
|  | Heart failure after cardiac surgery |
|  | Pulmonary laceration |
|  | Bone marrow suppression after chemotherapy |
|  | Neonatal asphyxia |
|  | Bee stings (poisonous insect bites) |
|  | Common atrium |
|  | Airway obstruction due to inhalation or swallowing of sputum or foreign bodies |
|  | Mitral and aortic valve disorders |
|  | Injuries to persons in vehicle accidents |
|  | Neonatal hyperbilirubinemic encephalopathy |
|  | Gallbladder stones |
|  | Myoepithelial carcinoma |
|  | frontal bone fractures |
|  | Atrioventricular anomalous channel |
|  | Ventriculoperitoneal shunt dislocation |
|  | Incarcerated inguinal hernia |
|  | Heart Failure |
|  | Aortic stenosis |
|  | Intestinal rupture |
|  | Circumferential pancreas |
|  | Aortic stenosis |
|  | Hepatic insufficiency |
|  | Protein-losing enteropathy |
|  | Vomiting of blood |
|  | Langerhans cell histiocytosis |
|  | Brain bulge |
|  | Poisoning by ingestion of poisonous mushrooms |
|  | Coronary artery occlusion |
|  | Scalp hematoma |
|  | Hemophagocytic syndrome |
|  | Intestinal duplication malformation |
|  | Anaphylaxis |
|  | Subcutaneous nodules |
|  | Pulmonary abscess |
|  | Cerebrovascular disease |
|  | Abnormal pulmonary venous connection |
|  | Splenomegaly |
|  | Tricuspid valve disease |
|  | Neonatal ABO hemolysis |
|  | Massive alveolar emphysema |
|  | Laryngeal obstruction |
|  | Hidradenitis |
|  | cardia flaccid inability |
|  | Paraplegia |
|  | Cranial separation |
|  | periodic paralysis |
|  | Accidental drug poisoning and exposure to such drugs |
|  | Traumatic lower limb amputation |
|  | Pneumothorax |
|  | Short bowel syndrome (post-intestinal resection syndrome) |
|  | Hypertension grade 1 |
|  | Hyponatremia |
|  | Intestinal torsion |
|  | Ureteral diverticulum |
|  | Double outlet of right ventricle |
|  | Shock |
|  | Alcohol intoxication |
|  | Coma |
|  | Specific tricuspid valve disease |
|  | Neonatal bronchopulmonary dysplasia |
|  | Hereditary spherocytosis |
|  | Neonatal pathologic jaundice |
|  | Microcephaly |
|  | Adnexal torsion |
|  | Triadic rhythm [ventricular prematureness] |
|  | Intestinal perforation (non-traumatic) |
|  | Acute hemolytic anemia |
|  | Acute Renal Failure |
|  | Anemia |
|  | Cyanosis |
|  | Cerebral anoxia |
|  | Neonatal respiratory distress syndrome |
|  | Common bile duct obstruction |
|  | Increased intracranial pressure |
|  | Fecal occult blood |
|  | Torsion of the greater omentum with necrosis |
|  | Skin rash |
|  | Incarcerated inguinal hernia with obstruction |
|  | Specific pesticide toxic effects (crops) |
|  | Skin abscess |
|  | Neonatal hypoglycemia |
|  | Granuloma of the small intestine |
|  | Post-operative hypocardial discharge after precordial surgery |
|  | Gastrointestinal foreign body |
|  | Headache to be investigated |
|  | Lung shadow |
|  | hyperammonemia |
|  | Peritoneal effusion |
|  | Actinic nerve palsy |
|  | Carcinomatous cerebral leukomalacia |
|  | Neonatal intestinal obstruction |
|  | Carbon monoxide toxic effects |
|  | Cirrhosis of the liver |
|  | Hepatogenic heart disease |
|  | Otawara syndrome |
|  | Neonatal abdominal distention |
|  | Removal of internal fracture fixation device |
|  | Congestive heart failure |
|  | Abnormal renal function |
|  | Cerebrovascular arteriovenous malformation |
|  | Gallbladder polyps |
|  | Behçet's syndrome |
|  | Intentional self-poisoning and exposure to chemical agents and harmful substances |
|  | Portal hypertension |
|  | Acute febrile mucocutaneous lymph node syndrome (Kawasaki disease) |
|  | Feeding disorders in infancy and childhood |
|  | Specific generalized epilepsy and epilepsy syndrome |
|  | Parapharyngeal abscess |
|  | Acute liver failure |
|  | Crohn's disease |
|  | Bile duct atresia |
|  | Uroplasia |
|  | Sciatic fracture |
|  | Intentional self-poisoning by drugs and exposure to such drugs |
|  | Acute respiratory failure |
|  | Methylmalonic acidemia |
|  | Hypertrophic non-obstructive cardiomyopathy |
|  | Septic shock (not associated with organ failure) |
|  | Hand, foot, and mouth disease (suspected) |
|  | Toxic effects of pesticides |
|  | Constipation |
|  | Tracheal stenosis |
|  | Acquired brain malformation |
|  | Pulmonary insufflation (atelectasis) |
|  | Skull base fracture |
